# Measurement of left ventricular dimensions with contrast-enhanced three-dimensional cine imaging facilitated by *k-t *SENSE

**DOI:** 10.1186/1532-429X-10-27

**Published:** 2008-05-28

**Authors:** Neil Maredia, Sebastian Kozerke, Abdul Larghat, Nik Abidin, John P Greenwood, Peter Boesiger, Sven Plein

**Affiliations:** 1 Academic Unit of Cardiovascular Medicine, University of Leeds, Leeds, UK; 2 Institute for Biomedical Engineering, University and ETH Zurich, Zurich, Switzerland

## Abstract

**Aim:**

To compare three-dimensional (3D) *k-t *sensitivity encoded (*k-t *SENSE) cine cardiovascular magnetic resonance (CMR), before and after contrast administration, against standard 2D imaging for the assessment of left ventricular volumes and mass.

**Method:**

Twenty-six subjects (14 volunteers, 12 patients) underwent multiple breathhold 2D balanced turbo-field echo cine CMR in addition to *k-t *SENSE accelerated 3D imaging (acceleration factor 5; 5× *k-t *SENSE), performed before and after administration of a high-relaxivity gadolinium-based contrast agent (Gadobutrolum). *k-t *acceleration factors of 7 and 10 were also assessed in six volunteers. Left ventricular end diastolic volume (EDV), end systolic volume (ESV), mass, and ejection fraction (EF) were calculated for each method.

**Results:**

There was at least moderate agreement between the EDV, ESV, mass and EF calculated by 2D and 3D 5× *k-t *SENSE methods before contrast (concordance coefficients 0.92, 0.95, 0.97, 0.92, respectively). Agreement improved following contrast (concordance coefficients 0.97, 0.99, 0.98, 0.93, respectively). The 3D method underestimated all parameters compared to 2D (mean bias pre-contrast 6.1 ml, 0.6 ml, 3.5 g, 2.0% respectively). 3D image quality scores were significantly poorer than 2D, showing a non-significant trend to improvement following contrast administration. Parameters derived with *k-t *acceleration factors of 7 and 10 showed poorer agreement with 2D values.

**Conclusion:**

Left ventricular volumes and mass are reliably assessed using 3D 5× *k-t *SENSE accelerated CMR. Contrast administration further improves agreement between 5× *k-t *SENSE and 2D-derived measurements. *k-t *acceleration factors greater than 5, though feasible, produce poorer agreement with 2D values.

## Introduction

The assessment of ventricular volumes and mass is an essential component of any cardiovascular magnetic resonance (CMR) study. Numerous studies have shown that CMR provides the most accurate and reproducible measurements of left ventricular dimensions [1-7], as it is able to provide data with high spatial resolution and full cardiac coverage. Conventionally, volumetric measurements are derived from 8–12 contiguous two-dimensional (2D) sections aligned in the true left ventricular short axis orientation. Because of constraints imposed by the data acquisition speed of current pulse sequences, 2D acquisition is the standard method used to obtain these data. Data acquisition is separated into a series of breath holds, usually one or two slices per breath hold. It is also feasible in principle to acquire a three-dimensional (3D) data set with CMR which covers the whole heart in a single volume stack. 3D acquisition can reduce registration errors between slices and simplify planning. However, with conventional CMR methodology, compromises in spatial or temporal resolution have to be made in order to permit 3D acquisition.

The recently proposed k-space and time sensitivity encoding (*k-t *SENSE) method allows substantial acceleration of data acquisition by applying sparse sampling along the spatial frequency (*k*) and temporal (*t*) encoding axes [8-10]. The resulting signal aliasing in the reciprocal spatio-temporal frequency or *x-f *domain is resolved using a filter constructed from low-resolution training data (as with the *k-t *BLAST technique) but additionally in conjunction with coil sensitivity information (unlike *k-t *BLAST, which does not utilize sensitivity information from multiple coils) [9]. Optimized sampling patterns were employed in data acquisition [11].

The method has been successfully applied to accelerate 2D cine imaging and for assessment of regional function in dobutamine stress CMR with a 3D cine implementation [12]. It was the purpose of the current work to evaluate measurements of LV volumes and mass from 3D *k-t *SENSE-accelerated cine imaging in comparison with standard 2D acquisition in volunteers and patients. In addition, several *k-t *acceleration factors and the effect of contrast media administration on volumetric and image quality measures of 3D *k-t *SENSE acquisition were assessed.

## Methods

### Subjects

The study population consisted of 26 subjects. Fourteen were healthy volunteers (mean age 26.7, range 20–49, ten male) and twelve were patients with suspected or known coronary heart disease (mean age 56.2, range 41–73, all male). Exclusion criteria for the study were contraindications to CMR (incompatible metallic implants, claustrophobia). All subjects gave written informed consent and the study was approved by the local ethics review board.

### CMR

CMR studies were carried out on a clinical 1.5 T MR system (Philips Medical Systems, Best, The Netherlands) using a 5 element cardiac phased array receiver coil for signal reception. Data were acquired during breath-holding in the end-expiratory position. Following scout images to determine the true short axis of the heart, breath-hold cine images in the axial, two-chamber and four-chamber planes were acquired to allow planning of the short-axis orientation. A multiple slice two-dimensional multiphase data set covering the LV in 10–12 short axis slices from apex to base was then acquired, which served as the reference study. The pulse sequence parameters for this reference study were as follows: Balanced steady state free precession (SSFP) pulse sequence (TR 2.8 ms, TE 1.4 ms, flip angle 55°, matrix size 256 × 256, spatial resolution 2.0 mm × 2.0 mm × 10 mm, no interslice gap, 20 phases/cardiac cycle, no parallel imaging, 1 slice per breath-hold, typical breath-hold 14 seconds per slice).

Subsequently, two identical *k-t *SENSE accelerated 3D data sets were acquired. The first was acquired prior to and the second immediately following the administration of an intravenous bolus injection of 0.1 mmol/kg bodyweight Gadobutrolum (Gadovist, Bayer Schering Pharma, Berlin, Germany). The scan orientation and spatial coverage were identical to the 2D reference study. The following parameters describe the pulse sequence used: 3D balanced steady state free precession (TR 3.2 ms, TE 1.6 ms, flip angle 50°, spatial resolution 2 mm × 2 mm × 10 mm, reconstructed to twelve sections, 20 phases/cardiac cycle, single breath-hold, *k-t *undersampling in two spatial dimensions, acceleration factor of 5, (5× *k-t *SENSE). The acquisition time was 14 seconds followed by a separate four-second breath-hold in which 49 training profiles were acquired. Taking into account the acquisition of training data, the effective acceleration factor achieved using the technique was 3.7. For the reconstruction of all *k-t *SENSE accelerated data, central k-space lines were substituted with training data ("training plug in") [10].

In six volunteers, the acquisition was repeated with *k-t *acceleration factor of 7 and 10 (7× *k-t *SENSE and 10× *k-t *SENSE) using otherwise identical scan parameters as outlined above. Acquisition times were 11 and 9 seconds for *k-t *acceleration factors of 7 and 10 respectively, each followed by a separate four-second breathhold for training profile acquisition.

#### Data Analysis

All data sets were analysed with commercially available analysis software (MASS, Medis, Leiden, The Netherlands) by an observer blinded to all patient information. 2D data sets were analysed initially, followed two weeks later by the 3D data sets in order to ensure blinding to the reference data.

Image quality, based on the ability to identify the endocardial border, was scored on a scale from 1 to 5, as follows: (1) endocardial border not visible (non-diagnostic image), (2) severe blurring of endocardial border, (3) moderate blurring, (4) mild blurring, (5) well defined endocardial border (modified from McConnell et al [13]). Artefacts were recorded with a score from 0 to 3, as follows: (0) no artefact, (1) minor artefact, (2) significant artefact but not affecting endocardial border definition, (3) significant artefact obscuring endocardial border in places.

On each data set, the endocardial and epicardial contours were traced manually at end-diastole and end-systole. The first phase of each slice was defined as end-diastole. End-systole was taken as the phase with the smallest total LV volume. LV end-diastolic volume (EDV) and LV end-systolic volume (ESV) were computed using the summation of discs method. Ejection fraction was determined as [(EDV-ESV)/EDV] × 100%. Left ventricular mass (LV mass) was calculated from the diastolic phase as LV mass = 1.05 g/cm^3 ^× (epicardial volume-endocardial volume).

#### Statistical Analysis

Statistical analysis was performed using SPSS version 15.0. Mean values +/- SD were calculated for all contour-related measurements. The mean bias between the methods and their limits of agreement were calculated according to the method described by Bland & Altman [14]. Lin's concordance coefficient [15] was used to assess the agreement between measurements obtained using different pulse sequences. This calculation was performed using the online concordance calculator hosted by the New Zealand National Institute of Water and Atmospheric Research (NIWA) [16]. The following scale was used to describe the strength of agreement: >0.99 almost perfect, 0.95–0.99 substantial, 0.90–0.95 moderate and <0.90 poor agreement. Image quality and artefact scores were compared using the non-parametric Wilcoxon signed ranks test.

The reproducibility of left ventricular volume assessment for each technique was assessed using a sample of ten participants (five volunteers and five patients). In order to assess intraobserver variability, left ventricular contours were redrawn several months after the original contours, and without reference to them, by the original observer (AL). An additional observer (NM) independently drew contours using the same images in order to assess interobserver variability. The error between repeated measurements was assessed in the manner described by Bland and Altman [17]. It was firstly confirmed that the degree of error was not proportional to the magnitude of the measurements, using Kendall's correlation coefficient and scatterplots. The variance between repeated measurements of EDV, ESV, EF and LV mass was then calculated for each subject. The within-subject variance for each parameter was obtained by taking the mean of the ten subjects' variances. The within-subject standard deviation was then derived by performing the square root of the within-subject variance.

## Results

**Figure 1:**
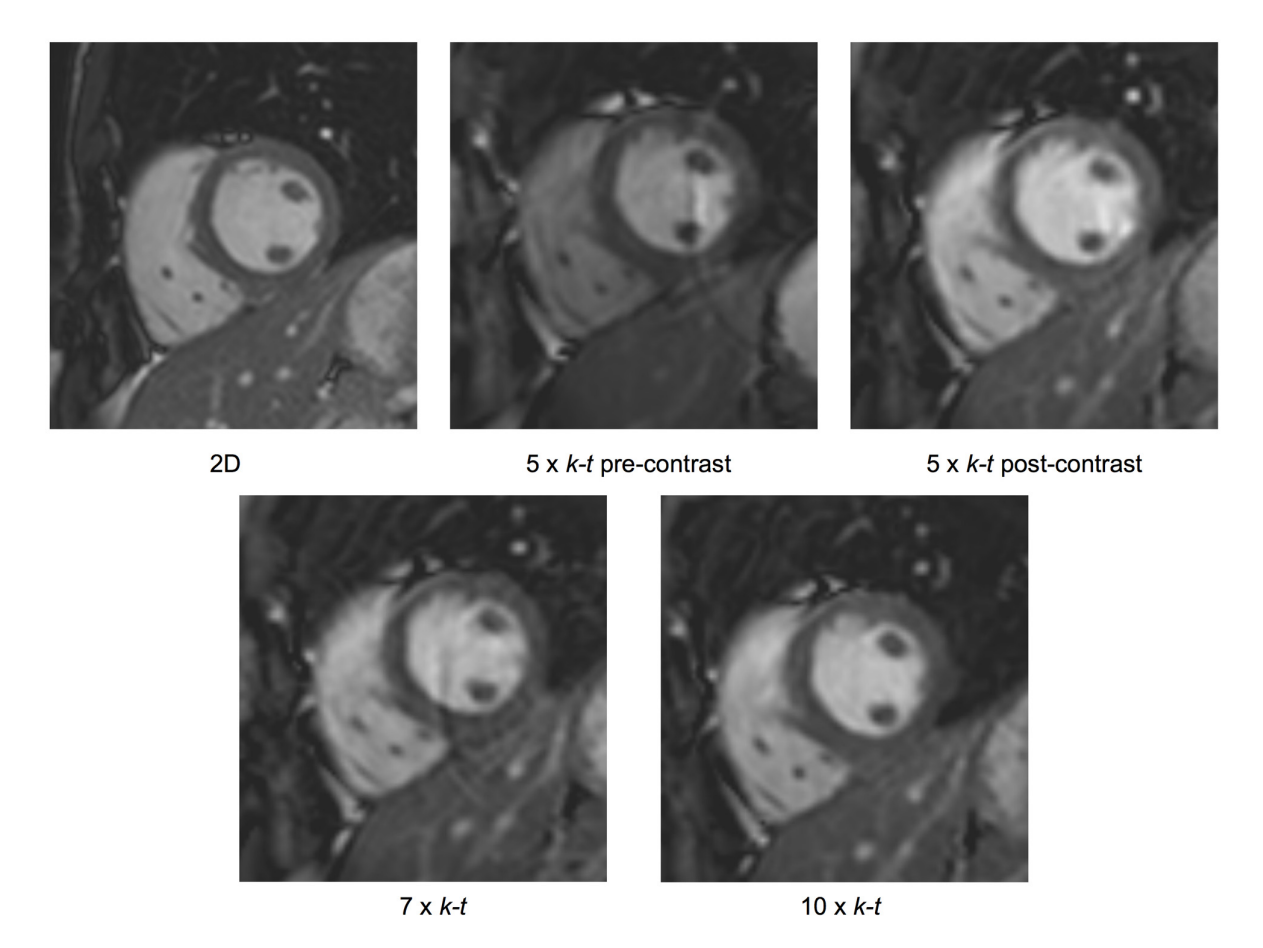
Sample images obtained from a single patient using each imaging technique.

Data acquisition was successfully completed in all subjects. Figure 1 demonstrates the images obtained from a single patient using each pulse sequence. The corresponding cine acquisitions can be viewed in movies 1–5.

### Image quality

**Figure 2:**
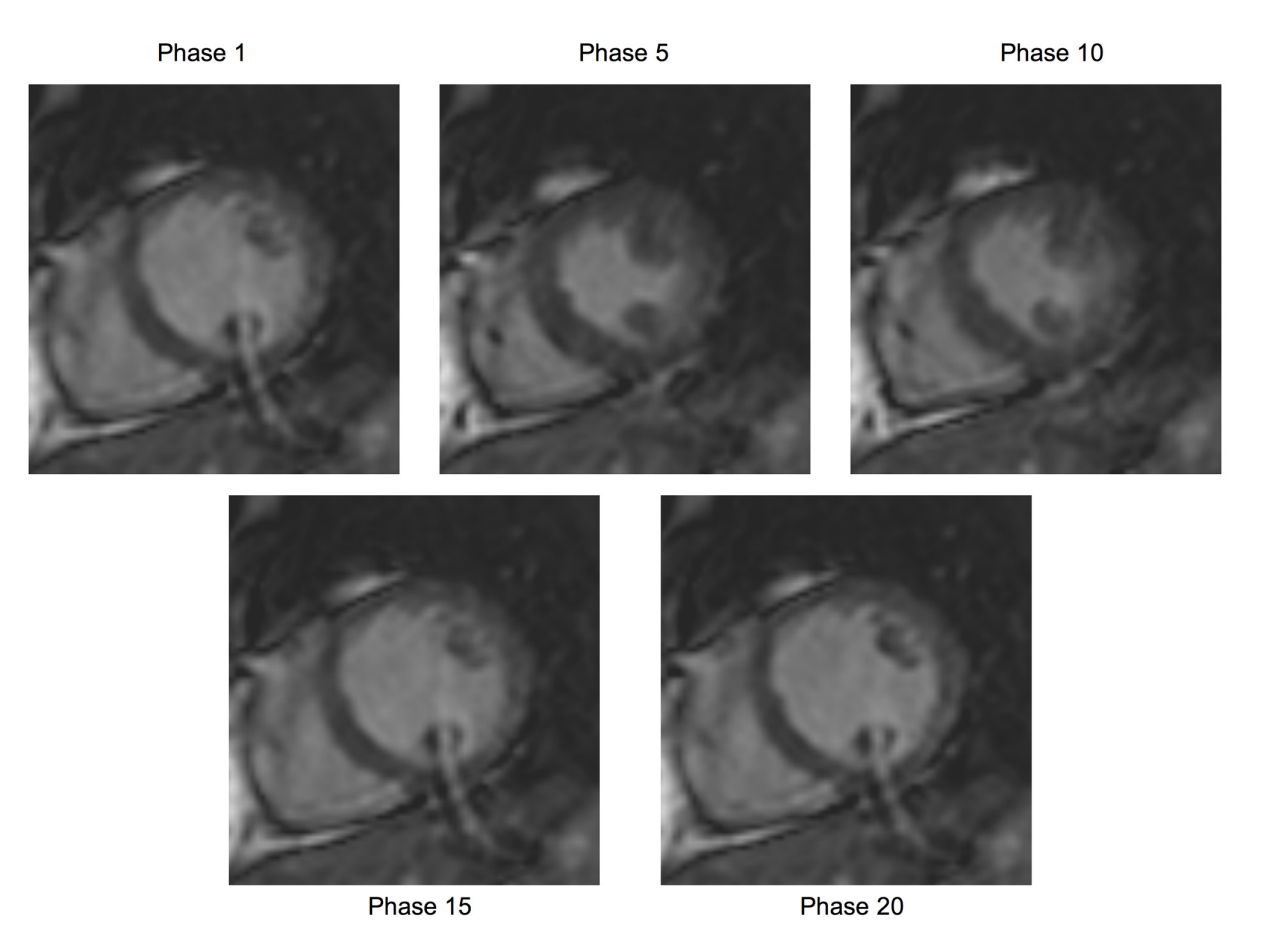
5× *k-t *SENSE accelerated images showing multiple phases from a single slice, illustrating a curved artefact, caused by aliasing of the fat signal along the slice direction. The severity of the artefact varies between different phases as a result of the different motion states of the heart.

The image quality scores of the 3D acquisitions were lower than the 2D reference images irrespective of the administration of contrast (table 1). Median image quality and artefact scores suggested improvements in the 3D post-contrast images when compared with the 3D pre-contrast images, but these differences were not statistically significant (pre-versus post-contrast image quality score p = 0.11, artefact score p = 0.23). The *k-t *accelerated images were noted to suffer from some temporal blurring and a curved artefact, which varied in intensity between phases (figure 2 and movies 2–5). This artefact is caused by aliasing of fat signal along the slice direction due to imperfect slice selection of the radio-frequency pulse in the general implementation of fast 3D sequences on the MR system we used.

**Table 1:** Image quality and artefact scores for the 2D reference and 3D 5× *k-t *SENSE images before and after contrast.

	Median Image Quality Score	Median Artefact Score
2D reference images	5	0

3D 5× *k-t *pre-contrast (p value vs 2D reference score)	3 (p < 0.01)	2 (p < 0.01)

3D 5× *k-t *post-contrast (p value vs 2D reference score) [p value vs pre-contrast 3D score]	4 (p < 0.01) [p = 0.11]	1 (p < 0.01) [p = 0.23]

Legend for Table 1

Image quality scoring: (1) endocardial border not visible (non-diagnostic image), (2) severe blurring of endocardial border, (3) moderate blurring, (4) mild blurring, (5) well defined endocardial border (modified from McConnell et al [13])

Artefact scoring: (0) no artefact, (1) minor artefact, (2) significant artefact but not affecting endocardial border definition, (3) significant artefact obscuring endocardial border in places

### Volumetric measurements

**Figure 3:**
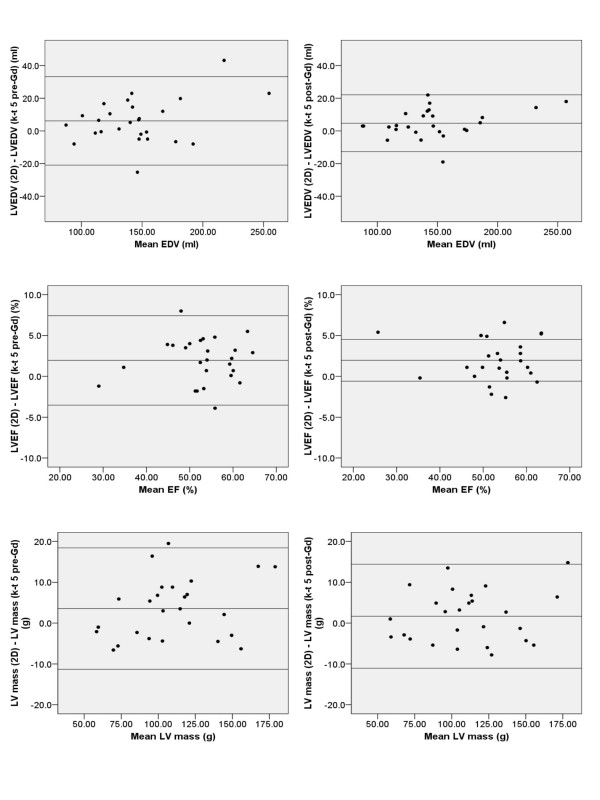
Bland Altman plots comparing EDV, mass and EF parameters obtained using the 5× accelerated *k-t *SENSE technique (k-t 5) before (pre-Gd) and after (post-Gd) contrast administration, against the 2D reference data. (The central horizontal line represents mean bias and the outer two horizontal lines represent the limits of agreement).

The results obtained using 2D and 3D 5× *k-t *SENSE (before and after contrast) are shown in table 2. Prior to contrast administration, the 3D 5× *k-t *SENSE technique demonstrated moderate agreement with 2D imaging for estimation of EDV and EF (concordance coefficients 0.92 for both), and substantial agreement for ESV and LV mass (concordance coefficients 0.95 and 0.97 respectively). The administration of contrast improved concordance coefficients for all parameters, producing substantial agreement for EDV, ESV and LV mass (concordance coefficients 0.97, 0.99 and 0.98 respectively) and moderate agreement for EF (concordance coefficient 0.93). This improvement is also reflected by a reduction in the limits of agreement for the bias between 2D and 3D 5× *k-t *SENSE for all parameters after contrast administration (figure 3).

**Table 2:** Comparison of 2D reference against 3D 5× *k-t *SENSE images before (pre-Gd) and after (post-Gd) contrast administration (n = 26)

	LV EDV (ml)	LV ESV (ml)	LV mass (g)	LVEF (%)
2D mean ± SD	149.0 ± 40.2	70.6 ± 33.7	111.7 ± 33.1	54.0 ± 8.3

3D 5× *k-t *pre-Gd mean ± SD	142.9 ± 35.4	70.0 ± 28.8	108.2 ± 31.1	52.0 ± 8.1
Bias (95% C.I.)	6.1 (-20.9; 33.2)	0.6 (-19.0; 20.3)	3.5 (-11.3; 18.4)	2.0 (-3.5; 7.4)
Lin's coefficient (95% C.I.)	0.92 (0.87–0.98)	0.95 (0.92–0.98)	0.97 (0.94–0.99)	0.92 (0.86–0.98)

3D 5× *k-t *post-Gd mean ± SD	144.3 ± 37.4	71.4 ± 32.3	110.0 ± 32.2	52.0 ± 8.4
Bias (95% C.I.)	4.7 (-12.7; 22.1)	-0.8 (-10.8; 9.2)	1.7 (-11.0; 14.4)	2.0 (-3.1; 7.1)
Lin's coefficient (95% C.I.)	0.97 (0.94–0.99)	0.99 (0.98–1.00)	0.98 (0.96–1.00)	0.93 (0.87–0.98)

Left ventricular EDV, mass and EF measurements were mostly underestimated by the 3D methods, irrespective of contrast administration. However, left ventricular ESV was underestimated by the 3D pre-contrast dataset but overestimated following contrast administration (mean bias 1.1 ml pre-contrast, -0.6 ml post-contrast).

### Higher k-t acceleration factors

Acquisition with higher *k-t *acceleration factors of 7 and 10 was feasible. The results obtained using higher acceleration factors are shown in table 3. The majority of these results show poor agreement with the 2D reference (concordance coefficients < 0.90), apart from the LV ESV result for 7× *k-t *SENSE, which agrees substantially with the 2D value (concordance coefficient 0.96). However, the small sample size means that the confidence intervals are wide and the data should therefore be interpreted with caution.

**Table 3:** Comparison of 2D reference against 3D 7× *k-t *SENSE and 3D 10× *k-t *SENSE images (n = 6).

	LV EDV (ml)	LV ESV (ml)	LV mass (g)	LVEF (%)
2D mean ± SD	145.8 ± 25.9	63.3 ± 19.1	95.4 ± 18.1	57.2 ± 5.9

3D 7× *k-t *mean ± SD	138.3 ± 26.2	62.3 ± 20.5	90.9 ± 18.6	60.9 ± 10.7
Bias (95% C.I.)	7.4 (-14.6; 29.4)	1.1 (-12.8; 14.9)	4.4 (-14.4; 23.3)	-3.7 (-26.7; 19.4)
Lin's coefficient (95% C.I.)	0.87 (0.64–1.00)	0.96 (0.88–1.00)	0.84 (0.56–1.00)	0.88 (0.67–1.00)

3D 10× *k-t *mean ± SD	134.3 ± 25.8	63.9 ± 22.0	92.7 ± 17.2	53.7 ± 8.0
Bias (95% C.I.)	11.5 (-16.1; 39.0)	-0.6 (-20.1; 18.9)	2.7 (-13.5; 18.9)	3.6 (-1.7; 8.8)
Lin's coefficient (95% C.I.)	0.77 (0.41–1.00)	0.89 (0.69–1.00)	0.88 (0.67–1.00)	0.81 (0.58–1.00)

### Reproducibility of LV Assessment

As demonstrated in table 4, the inter- and intraobserver variability values were similar for the 2D and 3D pre- and post-contrast methods.

**Table 4:** Inter- and intra-observer variability calculated from a sample of five patients and five volunteers (SD = Standard Deviation).

		Inter-observer Variability (Within-subject SD)	Intra-observer Variability (Within-subject SD)
**2D**	EDV (ml)	5.9	10.6
	ESV (ml)	7.9	7.9
	LV mass (g)	12.0	9.4
	LV EF (%)	4.0	2.9

**3D 5× k-t Pre-Gd**	EDV (ml)	9.0	5.2
	ESV (ml)	6.1	5.9
	LV mass (g)	14.8	11.1
	LV EF (%)	2.4	3.5

**3D 5× k-t Post-Gd**	EDV (ml)	9.3	9.2
	ESV (ml)	7.3	8.1
	LV mass (g)	13.9	9.2
	LV EF (%)	3.5	3.8

## Discussion

This study shows that *k-t *SENSE-accelerated 3D CMR cines can be used to reliably assess left ventricular mass, ejection fraction, end systolic and end diastolic volumes.

Using the 5× *k-t *SENSE acceleration method presented here, a full LV study, with temporal and spatial resolution equivalent to the 2D reference method, could be acquired using a single 14-second breathhold followed by a 4-second breathhold for training data. This compares to several minutes for a full LV cine study using a conventional 2D approach. The differences observed between LV parameters derived from 2D and 3D *k-t *accelerated images in this study are sufficiently small to be irrelevant in most clinical scenarios. The rapid and reliable acquisition of left ventricular images through the 5× *k-t *SENSE acceleration method will be particularly of benefit when scanning unstable patients who are unable to tolerate long periods of time in the MR scanner.

The administration of T1 shortening contrast media reduced artefacts and improved the image quality scores of the 5× *k-t *SENSE accelerated images, though these differences did not prove statistically significant. However, the trend towards an improvement in image quality scores was mirrored by an improvement in the agreement between 2D and 3D volumetric measurements following contrast. In many CMR studies, contrast agents are administered for other components of the study, so that contrast-enhanced 3D cine imaging will not necessitate additional contrast administration. In most instances, conventional contrast agents, such as the one used in this work, will be chosen rather than intravascular agents. Although intravascular contrast has shown some advantages for 3D cine imaging and might further improve the delineation of the blood pool versus myocardium [18] our results using an extravascular agent are more relevant to current clinical practice.

It is interesting to note that, despite generally good agreement between the techniques, there was a systematic underestimation of LV volumes, ejection fraction and mass with the 3D methods compared with the 2D reference. There are several explanations for the differences between 2D and 3D acquisition results. During 3D imaging, a reduction in the inflow of unsaturated blood can reduce endocardial border definition and thereby influence contour placement. Furthermore, individual 2D slices are generally better defined than in 3D acquisition, where the reconstructed slices suffer from more partial volume effects due to the extent of the point-spread function in the slice selection direction. The appearance of the endocardial border therefore differs between the two methods. With 2D techniques, spatial misregistration can also affect volumetric measurements, although this would be expected to have a random effect. Finally, when prospective gating is used in data acquisition as used in the present study, the cardiac cycle may not be covered in its entirety, in particular if the actual heart rate differs from the anticipated heart rate. Because the *k-t *reconstruction algorithm assumes a cyclic temporal function, discontinuities between the last and first phases of the acquisition can lead to reconstruction errors in the first and last heart phase images. Since we defined EDV as the first phase of the acquisition, such errors may have contributed to its underestimation. With retrospective gating, which has recently been implemented for *k-t *SENSE [19], the potential for this error will be removed.

Two recent studies using a similar acceleration method, *k-t *BLAST, and prospective triggering, have shown similar results. A study of 40 patients by Jahnke et al has reported a mean bias for LVEDV of 4.9%, similar to the result of our study, with better agreement for ESV and EF [20]. Meanwhile, Greil et al, in their study of 17 volunteers, found similar results (mean difference for 6× *k-t *BLAST acceleration vs. 2D cine: EDV 5 ml, ESV 1 ml, LV mass -0.9 g, EF 0.5%), and further demonstrated the tendency to underestimation of left ventricular volumes using *k-t *acceleration [21]. The consistency in results between these three independent studies suggests a systematic error between 2D and 3D acquisition techniques, which may be related to the reconstruction algorithm used.

Traditional parallel imaging techniques are generally limited by decreasing signal to noise ratios (SNR) as acceleration factors increase. However, acceleration by *k-t *SENSE is significantly more SNR efficient. The main factors limiting the degree of acceleration with this technique are temporal blurring and associated artifacts. In this study, up to 10-fold *k-t *acceleration factors were tested and found to be feasible in a small group of volunteers. For 10-fold acceleration, data acquisition required only a nine-second breathhold followed by a four-second breathhold for training data. At these higher acceleration factors however, temporal blurring and artefacts were observed on qualitative analysis. Consequently, the measurements made using these images showed poor agreement with the 2D reference results.

## Limitations

The higher *k-t *acceleration factors of 7 and 10 were only tested in six volunteers. Though this establishes the feasibility of the technique, larger scale studies will be needed to determine whether our finding of poor agreement with the reference standard is reproduced.

## Conclusion

This study demonstrates that *k-t *SENSE accelerated 3D left ventricular cine imaging can be used to reliably measure most clinically important left ventricular parameters. Images produced using a *k-t *acceleration factor of 5 generally showed good agreement with the 2D reference, particularly after the administration of contrast agent. Measurements derived from images using higher *k-t *acceleration factors of 7 and 10, though proven to be feasible, showed poorer agreement with the reference technique. Further studies with larger groups of patients will be needed to determine the role of *k-t *SENSE accelerated 3D cine CMR imaging in routine clinical practice.

## Supplementary Material

Additional file 1 2D mid-ventricular slice cine. This movie shows a mid-ventricular slice cine, taken with a 2D sequence. Click here for file

Additional file 2 3D 5× *k-t *mid-ventricular slice cine, before contrast. This movie shows a mid-ventricular slice cine, taken with a 3D 5× *k-t *sequence, before the administration of contrast. Click here for file

Additional file 3 3D 5× *k-t *mid-ventricular slice cine, after contrast. This movie shows a mid-ventricular slice cine, taken with a 3D 5× *k-t *sequence, after the administration of contrast. Click here for file

Additional file 4 3D 7× *k-t *mid-ventricular slice cine. This movie shows a mid-ventricular slice cine, taken with a 3D 7× *k-t *sequence. Click here for file

Additional file 5 3D 10× *k-t *mid-ventricular slice cine. This movie shows a mid-ventricular slice cine, taken with a 3D 10× *k-t *sequence. Click here for file
